# Deanna Protocol Ameliorates the Deficits in Human iPSC‐Cortical Neurons Carrying Familial Alzheimer's Mutation

**DOI:** 10.1002/cns.71205

**Published:** 2026-10-23

**Authors:** Leticia Keler Soares, Gaurav Srivastava, Camden Dwelle, Brianna Waggoner, Vince Tedone, James J. Hickman, Xiufang Guo

**Affiliations:** ^1^ University of Central Florida Orlando Florida USA; ^2^ Hesperos Inc. Orlando Florida USA; ^3^ WFND Tampa Florida USA

**Keywords:** Alzheimer's disease, cellular energy, cortical neurons, Deanna Protocol, iPSC, long‐term potentiation, microelectrode array

## Abstract

**Aims:**

Deanna Protocol (DP) is an anaplerotic, mechanism‐based therapy designed to address impaired cellular energy flux. The present study aims to investigate the effects of DP in treating Alzheimer's disease (AD) by using cortical neurons derived from AD patient iPSCs carrying a *PSEN1* mutation, while cortical neurons differentiated from iPSCs of a healthy subject serve as control.

**Methods:**

*PSEN1* cortical neurons were treated with DP acutely (24 h) and chronically (1–3 weeks). The effect of DP was assessed by utilizing TMRE assay for mitochondrial function, immunofluorescent staining for AD neuron morphological pathology, whole cell patch clamp for neuron electrophysiological property, and microelectrode array (MEA) analysis of long‐term potentiation (LTP) for neurocircuit function.

**Results:**

*PSEN1* cortical neurons demonstrated impaired mitochondrial function, elevated pathological biomarkers, reduced neurite complexity, altered electrophysiology, and decreased LTP. DP treatment significantly ameliorated all these phenotypes.

**Conclusion:**

This study, while confirming iPSC‐*PSEN1* cortical neurons as a valid AD model, demonstrated the efficacy of DP on ameliorating structural and functional pathologies in AD neurons. It highlights that targeting energy production could be an effective approach in treating neurodegeneration in AD and suggests its potential efficacy in other neurodegenerative diseases.

## Graphical abstract

Human iPSC‐derived cortical neurons carrying a *PSEN1* Alzheimer's mutation exhibit mitochondrial dysfunction, hyperexcitability, amyloid and tau pathology, and impaired synaptic plasticity. The Deanna Protocol, an anaplerotic metabolic supplement, rescues these deficits, supporting energy restoration as a viable therapeutic strategy for Alzheimer's disease.

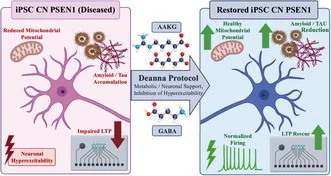

## 1 Introduction

Alzheimer's disease (AD) is a progressive neurodegenerative disorder (ND) and the most common cause of dementia worldwide. It is characterized by the extracellular bioaccumulation of amyloid‐β (Aβ) plaques and intracellular aggregation of neurofibrillary tangles composed of hyperphosphorylated tau, leading to synaptic loss and gradual neurodegeneration [1]. Familial forms of AD arise from autosomal dominant mutations in genes such as *PSEN1*, *PSEN2*, and *APP*, which disrupt Aβ processing and enhance pathogenic Aβ species, particularly the Aβ42/40 ratio [2, 3]. Among these mutations, *PSEN1* mutations are the most common, accounting for approximately 75% of genotyped families positive for a mutation and are often associated with aggressive early‐onset disease, which offers insights into the AD pathogenesis [4, 5].

A critical barrier to understanding AD mechanisms and developing interventions is the limited translational relevance of traditional animal and immortalized models. Although they have advanced our knowledge, they often lack the intricate human‐specific genetic and physiological context, especially in the case of familial AD mutations. Human induced pluripotent stem cell (iPSC) technology can bridge this gap by generating neurons that retain the donor's genetic background, including disease‐causing mutations, enabling the modeling of disease‐specific cellular and molecular phenotypes [6]. iPSC‐derived cortical neurons (iPSC CNs) represent a physiologically relevant system for studying AD‐related functional and pathological changes, such as electrophysiological activity, synaptic dysfunction, and tau hyperphosphorylation [7]. Notably, iPSC models carrying *PSEN1* mutations resemble key features of familial AD, including elevated Aβ42 levels, tau pathology, disruptions in synaptic signaling, and mitochondrial function [8, 9, 10].

Energy depletion, primarily from mitochondrial dysfunction and impaired glucose metabolism, is recognized as a central mechanism in neurodegenerative diseases, leading to synaptic failure, buildup of protein aggregates (like Aβ and tau), oxidative stress, inflammation, and ultimately cell death [10, 11]. Therefore, targeting energy deficiency could be an effective therapy for neurodegenerative diseases. Mitochondrial dysfunction and impaired energy metabolism are recognized hallmarks of AD pathology and contribute to neuronal degeneration and tau hyperphosphorylation [12]. The Deanna Protocol (DP) is a metabolic supplement regimen—developed initially for amyotrophic lateral sclerosis (ALS)—that includes initially components such as arginine‐alpha‐ketoglutarate (AAKG), coenzyme Q10, medium‐chain triglycerides, and GABA analogs, designed to bypass impaired energy pathways and support mitochondrial function [13]. The positive effect of DP on mitochondrial function and cellular metabolism, as well as its therapeutic effect on ALS, has been documented in anecdotal reports from ALS patients [13], and demonstrated in a SOD1‐G93A mouse model [13, 14]. The therapeutic effect of DP was further confirmed in a human iPSC‐motoneuron ALS model by analyzing their effect on ameliorating axonal pathology induced by glutamate or ALS mutations [15], and in an iPSC‐derived ALS neuromuscular junction (NMJ) model through rescuing NMJ function impaired by ALS mutations [16]. The composition of the DP was progressively optimized along with efficacy testing in different preclinical ALS models and anecdotal clinical evaluation, with AAKG and GABA consistently being the core compounds.

With the well documented effect of DP on mitochondria and cellular metabolism and ALS disease, this study aimed to determine whether DP has a therapeutic effect on AD, a different neurodegenerative disease which shares the impairment of cellular metabolism. Specifically, the effect of DP on repairing the electrophysiological functional deficits and reducing the cellular pathology (Amyloid and Tau) will be investigated in a human‐relevant iPSC CN AD model. The outcome will inform future therapeutic strategies.

## 2 Results

The DP utilized in this study is the commercially available product, composed of the core DP components, AAKG and GABA formulated in a proprietary composition. The effect of DP on the morphological and molecular characteristics, mitochondrial integrity and neuronal function was analyzed systematically in human iPSC CNss differentiated from both wild‐type and *PSEN1* ^*A246E*^‐mutant iPSC lines. Following acute (24‐h) and chronic (1–3‐week) DP treatment, mitochondria function was analyzed by TMRE assay. Neuronal excitability and network activity were assessed by MEA recordings and whole‐cell patch‐clamp electrophysiology analysis. Additionally, immunocytochemistry (ICC) for MAP2, neurofilament (NF), amyloid β (Aβ) deposition, and phospho‐tau (P‐tau) provided insights into structural pathological modulation. For chronic treatment, the description by week(s) has accommodated +1 day approximation, that is, 1 week corresponds to 8 days, 2 weeks to 15 days, and so forth. The choice of the timing and duration of the treatment is based on the properties of the readouts and the longitudinal features of the cell culture.

### 2.1 DP Enhanced Mitochondrial Function in *PSEN1* Cortical Neurons

Tetramethylrhodamine ethyl ester (TMRE) fluorescence was used as a functional readout of mitochondrial membrane potential (MMP) and energetic capacity in WT and *PSEN1* ^*A246E*^ iPSC CNs after DP treatment (Figure 1). On either DIV21 (after 24 h DP) or DIV28 (after 1 week of DP), *PSEN1* DP− neurons exhibited lower TMRE signals compared to WT controls, in line with the mitochondrial dysfunction and oxidative stress that is well recognized in AD neurons [17, 18] and causes ATP deficiency. DP treatment significantly increased TMRE fluorescence in *PSEN1* neurons, suggesting improved mitochondrial function. Interestingly, TMRE signal in WT neurons was reduced after DP treatment, and a slightly greater reduction in DIV28 than in DIV21, but never reaching PSEN1 level. This may correlate to the status of enhanced ATP production due to the increased supply of TCA intermediates, which released the proton gradient causing MMP reduction. This differential hypothesis for PSEN1 and WT neurons is further supported by the structural and functional in following analysis.

**FIGURE 1:**
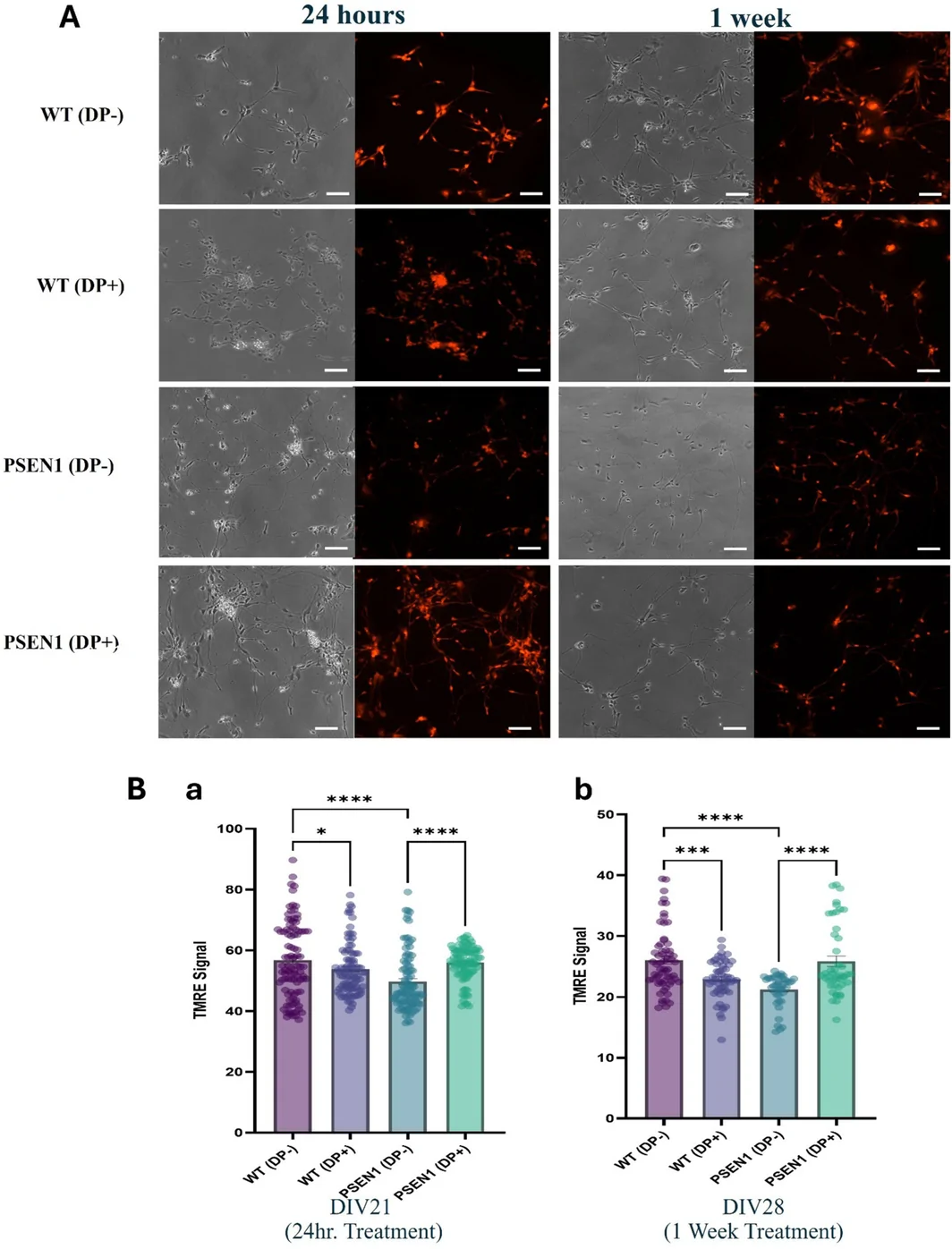
Evaluation of DP effect on mitochondria function by TMRE analysis. (A) Sample TMRE images from each condition. (B) Mean TMRE signal quantified on (a) DIV 21 CNs after 24‐h DP treatment, and (b) DIV 28 CNs after 1‐week DP treatment. Data represents mean + SEM. For each condition, more than 90 neurons from three batches of experiments, one coverslip per batch were analyzed. Two‐way ANOVA. *p < 0.05; ***p < 0.001; ****p < 0.0001.

### 2.2 Prolonged DP Treatment Increased NF Intensity and Attenuates Tau Phosphorylation in *PSEN1* Cortical Neurons

To evaluate the effects of DP on neurofilament (NF) and tau pathology, immunocytochemical analysis was conducted at DIV35 in WT and *PSEN1* iPSC CNs after 3 weeks of treatment. Quantification of NF showed that *PSEN1* neurons had significantly lower signal intensity than WT neurons. DP treatment significantly increased NF intensity in both WT and *PSEN1* neurons, suggesting that DP may partially stabilize or preserve axonal integrity in neurons in general (Figure 2B,i). This is different from 1 week treatment (Figure S1A) in which the increase did not show statistical significance. This difference presumably reflects the cumulative effect of DP on preserving neuron axonal structure after prolonged treatment. Analysis of P‐tau immunoreactivity indicated significantly higher levels in *PSEN1* DP‐ neurons compared to WT, which was visibly reduced by DP treatment, same for both 1 and 3 weeks of treatment. This reduction remained significant when normalized to NF, indicating that decreased tau phosphorylation was not attributable to loss of axonal processes (Figures 2B,ii,iii and S1B,C). WT neurons displayed low P‐tau levels, which were unaffected by treatment.

### 2.3 DP Decreased Amyloid Aggregation in *PSEN1* Cortical Neurons

To assess the effects of prolonged DP treatment on Aβ aggregation, a hall marker for AD, immunocytochemical analysis was conducted at DIV35, 3 weeks after DP treatment. Neuronal dendritic structure was evaluated using MAP2 staining, and amyloid aggregation‐associated signal was measured using Methoxy‐X04 labeling (Figure 3). Representative confocal images demonstrated increased Methoxy‐X04 signal in *PSEN1* DP− neurons compared to WT cultures. Conversely, DP‐treated *PSEN1* neurons showed visibly reduced Methoxy‐X04 staining. WT neurons maintained low Methoxy‐X04 signal across all treatment conditions (Figure 3A). Quantification of MAP2 fluorescence intensity revealed reduced MAP2 signal in *PSEN1* DP− neurons compared to WT cultures (Figure 3B,i). DP treatment increased MAP2 intensity in *PSEN1* neurons, and slightly in WT neurons, indicating a better preserved or increased dendritic structure. Methoxy‐X04 fluorescence quantification demonstrated a significant increase in Aβ aggregation in *PSEN1* cultures, which was significantly reduced by DP treatment (Figure 3B,ii). This reduction remained significant when normalized to MAP2, indicating that the decrease in Aβ signal was not attributable to lower dendritic processes (Figure 3B,iii). WT neurons maintained low Methoxy‐X04 levels across all conditions. This analysis demonstrates that DP treatment reduced Amyloid accumulation in *PSEN1* cortical neuron cultures while preserving dendritic integrity.

### 2.4 DP Reduced Hyperexcitability of *PSEN1* Cortical Neurons Analyzed by Patch Clamp Electrophysiology

Cortical hyperexcitability, a state of neuronal overactivity due to reduced inhibition, is a key early pathological feature of AD [19]. In correlation, early stage AD patients prone to experience increased seizures, which seems to facilitate Aβ and tau deposition, thus promoting neurodegenerative processes [20]. To investigate the effect of DP on neuronal excitability, DIV21 cortical neurons were analyzed by patch clamp electrophysiology after 1 week of DP treatment. The analysis for spontaneous firing and repetitive firing was illustrated in Figure 4 for quantitative comparison and in Figure S2 for sample traces. The result for other parameters was included in Figure S3. Compared to WT neurons, *PSEN1* neurons demonstrated higher spontaneous firing activity (Figure 4A,C), and increased repetitive firing upon depolarization (Figure 4B,D). DP treatment reduced both although but did not reach statistical significance. Both spontaneous firing and induced repetitive firing activity in WT neurons were not significantly affected by DP. Quantification of recorded neurons indicated that all the *PSEN1* CNs displayed spontaneous firing (100% in *PSEN1* vs. 50% in WT). Additionally, *PSEN1* CNs frequently exhibited bursting firing pattern recorded in spontaneous firing (Figure 4E), which is usually a sign of hyperexcitability. It was also observed in WT CNs but at lower percentage (54.54% in PSEN1 vs. 24.24% in WT). DP treatment reduced the number of neurons showing bursting firing in both WT and *PSEN1*.

### 2.5 DP Potentiate Network Plasticity in *PSEN1* Cortical Neurons

The effect of DP on populational neural activity and neural circuit function was evaluated using microelectrode array (MEA) analysis. Long‐term potentiation (LTP), a cellular surrogate for learning and memory, was analyzed. As in Figure 5, cortical neurons were cultured on paired electrodes following surface patterns on MEA. LTP was induced with high‐frequency stimulation (HFS) following our established protocol [21, 22], and the neural activity recorded at 1 and 24‐h post HFS was analyzed, named 1‐h LTP, 24‐h LTP, respectively. Effect of DP on LTP induction was analyzed after 24 h (DIV21), 1 week (DIV28), and 2 weeks (DIV35) treatment.

The result of DIV35 was demonstrated in detail in Figure 5C,D, while those for DIV21 and DIV28 were demonstrated in Supplement (Figures S4 and S5, respectively). After HFS, increased firing frequency was observed right after (post‐HFS), 1 and 24 h in WT system and WT treated with DP (Figure 5D). On the contrary in *PSEN1* system, HFS did not induce increased activity, but significant reduction of firing frequency at 1‐h post‐HFS (Figure 5D,c). Failure to respond to HFS and the impairment of LTP indicates a deficit in neuronal function and synaptic plasticity. DP treatment increased both 1‐h and 24‐h LTP in *PSEN1* systems while the increase of 24‐LTP was statistically significant (Figure 5D,d). For all these MEA experiments, lidocaine treatment at the end of recordings silenced all the neuronal firing, confirming the biological source of the recorded signals.

To analyze the longitudinal phenotypes of *PSEN1* cortical network and effect of DP after different periods of DP treatment, the systems were analyzed at on DIV21 and DIV28. On DIV21 (Figure S4), no LTP was induced in WT systems, suggesting an immature developmental stage, similar to our previous publication [22]. DP treatment induced a significant increase of 24‐h LTP in WT systems, suggesting a potentiation of network function. In PSEN1 systems, increased 1‐h and 24‐h LTP were observed, in which 24‐h LTP reaches statistical significance. This result aligns with the hyperexcitability phenotype observed on DIV21 *PSEN1* neurons in patch clamp. On DIV28 (Figure S5), WT systems demonstrated increased firing frequency at all the tested timepoints after HFS, in which post‐HFS and 1‐h LTP reach statistical significance, suggesting gradual functional maturation. However, *PSEN1* systems did not show overall increase of firing frequency after HFS at any time point tested, indicating a pre‐mature functional decline. Horizontal comparison of LTP amplitude across the four experimental groups (WT, WT + DP, *PSEN1*, and *PSEN1* + DP) on DIV21, DIV28, and DIV35 better demonstrated the trends of pathology and DP effect (Figure 6). On DIV21, *PSEN1* systems demonstrated higher LTP amplitude than WT systems, in which the increase of 24‐h LTP reaches statistical significance, aligning with the hyperexcitability phenotypes. On DIV28, *PSEN1* CNs demonstrated reduced LTP than WT, in which the reduction of 1‐h LTP was statistically significant. One week of DP treatment significantly increased both 1‐ and 24‐h LTP in *PSEN1* systems. On DIV35, the LTP reduction in *PSEN1* systems became statistically significant for both 1‐ and 24‐h LTP, which were both effectively corrected after 2 weeks of DP treatment. This longitudinal LTP analysis demonstrated the development of *PSEN1* functional phenotype over time, which started with early stage hyperexcitability then gradually declined prematurely. This trend mirrors the clinical profiles during disease progression, which reports epilepsy in early stages followed by premature functional decline as neurodegeneration progresses [20, 23]. This functional decline is significantly mitigated by DP treatment.

## 3 Discussion

This work investigated the effect of DP on rescuing the deficits of iPSC‐derived cortical neurons harboring *PSEN1* ^*A246E*^ mutation. The AD neurons demonstrated reduced NF‐positive structures, increased amyloid deposition and P‐tau by morphological analysis, reduced mitochondria membrane potential, hyperexcitability, and defective LTP induction. DP treatment demonstrated positive effects on almost all the phenotypes.

DP consists of three components: alpha‐ketoglutarate (AKG), l‐arginine (l‐Arg), and GABA. AKG, an intermediate of the TCA cycle, is involved in pleiotropic metabolic and regulatory pathways in the cell, including energy production, biosynthesis of certain amino acids, collagen biosynthesis, epigenetic regulation of gene expression, regulation of redox homeostasis, and detoxification of hazardous substances [24]. AKG supplement was found to extend lifespan and health span, compressed morbidity, and decrease the levels of systemic inflammatory cytokines in aging mice models [25]. In general, reconstitution of AKG supplementation directly restores oxidative phosphorylation by feeding carbon into the TCA cycle. l‐Arg, a bipolar base, was added to neutralize acidic AKG while adding other benefits. As a semi‐essential amino acid, l‐Arg has emerged as a promising therapeutic candidate for neurodegenerative diseases due to its ability to act as a chemical chaperone that prevents protein misfolding and its role as a precursor for nitric oxide (NO), which has neuroprotective function in the CNS [26, 27, 28]. l‐Arg also reduces oxidative stress by scavenging free radicals and lowers the expression of proinflammatory cytokines linked to neuroinflammation [29]. Additionally, Arginine enhances oral tolerance and augments blood perfusion through NO synthesis. The inclusion of the inhibitory neurotransmitter, GABA, helps to counteract the excitotoxicity and restore the excitatory‐inhibitory balance. Excitotoxicity has been recognized as a pivotal player in the pathogenesis of AD and other neurodegenerative diseases and has been proposed as an effective therapeutic target [30, 31, 32]. Biochemically, the GABA shunt is a carbon‐entry (anaplerotic) route into the TCA cycle because GABA's 4‐carbon backbone is converted to succinate, a bona‐fide TCA intermediate, thereby supporting mitochondrial energy production [33]. Together, these actions re‐establish energy homeostasis and synaptic integrity, which are neuroprotective across general degenerative conditions.

It is hypothesized that metabolic malfunction is a central pathogenic event shared across NDs, in which a redirection of glycolytic flux from pyruvate into lactate results in functional disconnection of glycolysis from the tricarboxylic acid (TCA) cycle [34, 35, 36]. Multiple studies documented reduced activity of key TCA enzymes (pyruvate dehydrogenase complex [PDH], α‐ketoglutarate dehydrogenase [α‐KGDHc]), depletion of TCA intermediates, and failure of glucose oxidation, leading to neuronal energy failure [37, 38, 39, 40]. This metabolic bottleneck is under‐investigated relative to downstream proteinopathies and represents a rational target for metabolic/anaplerotic repair. Under physiologic conditions, pyruvate enters mitochondria and is oxidatively decarboxylated by PDH to form acetyl‐CoA, initiating the TCA cycle and sustaining ATP generation. In ND, however, PDH inhibition, NAD^+^ depletion, and oxidative stress divert pyruvate toward lactate formation via lactate dehydrogenase‐5 (LDH5). This metabolic rerouting mirrors the Warburg phenomenon observed in neoplasia—an aerobic glycolysis producing lactic acid despite adequate oxygen—and results in profound energetic insufficiency [41]. Elevated brain lactate and reduced oxidative phosphorylation have been demonstrated in post‐mortem and in vivo studies across AD, Parkinson's disease, ALS, and Huntington's disease [41, 42]. This TCA‐to‐lactate shifting in cell metabolism in ND provides the rationale for anaplerotic treatment strategies, which is echoed by a recent study in mice indicating restoring NAD^+^ homeostasis reversed advance AD pathology [43]. Particularly, α‐KGDHc, a rate‐limiting enzyme in the TCA cycle, has significantly reduced activity in AD brains, contributing to cerebral energy metabolism loss, and correlated with cognitive decline, amyloid, and tau pathology [37, 38]. Supplementation of AKG in DP, the substrate of α‐KGDHc, would compensate for the inefficiency of this enzyme and promote ATP production. Additionally, metabolic acidosis caused by lactate accumulation will potentiate the activity of Na^+^/dicarboxylate cotransporters, the key transporters for the cellular uptake of AKG, and increase AKG uptake [44]. Therefore, AKG supply under these pathological conditions provides attainable treatment for the cells under metabolic stress.

When neuronal metabolism is restricted to glycolysis ending in lactate, ATP output falls dramatically. Consequently, neurons lose the ability to maintain the Na^+^/K^+^ gradient, vesicular cycling, and excitatory–inhibitory balance, leading to hyperexcitability at early stage but later synaptic silencing. Similarly, the clearance of excitatory neurotransmitter, glutamate, from extracellular space would be less efficient in the lack of ATP due to the compromises or even reversal of ATP‐consuming Na^+^−dependent glutamate transporters [45, 46, 47], which will further exacerbate the excitotoxicity [48]. Patch clamp analysis in this study well illustrated the increased spontaneous firing and repetitive firing under stepwise depolarization at DIV 15 and DIV 21 in *PSEN1* CNs, which are signs of hyperexcitability. DP treatment effectively suppressed this phenotype (Figure 4). Further MEA studies confirmed the hyperexcitability phenotype in *PSEN1* CNs on DIV 21, manifested as higher LTP amplitude (Figure 6). However, reduced LTP on DIV 28 indicated impaired synaptic plasticity as the neurons mature or age, which was mitigated or reversed by DP treatment. These observations align well with the hypothesis of energy deficiency in *PSEN1* neurons. Simultaneously, energy deficiency can cause the malfunction of protein processing and accumulation of intracellular peptides and misfolded proteins, where they aggregate into amyloid plaques and neurofibrillary tangles, hallmarks of AD and related tauopathies. These projected structural pathologies have been well illustrated in *PSEN1* CNs through extensive morphological analysis, which are significantly ameliorated by DP (Figures 2 and 3). All these results support that energy deficiency is a significant driver and effective therapeutic target for neurodegeneration in AD.

**FIGURE 2:**
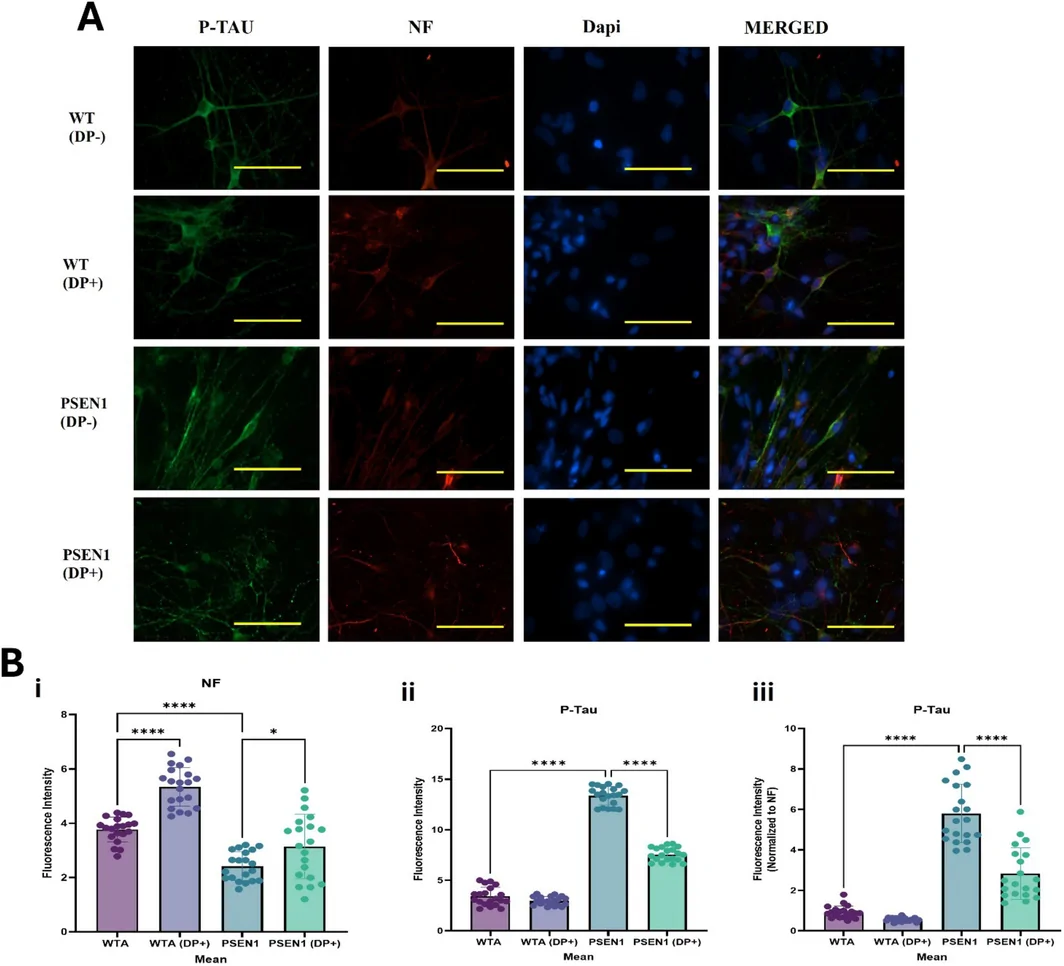
Evaluation of DP effect on NF and Phospho‐tau (P‐tau 181) by immunocytochemistry in cortical neurons (DIV 35) after 3 weeks of DP treatment. (A) Confocal images of WT and *PSEN1* CNs stained with P‐tau (green) and NF (red). Scale bar: 100 μm. (B) Quantification of the mean intensity of the images for the signals of: (i) NF; (ii) P‐tau; and (iii) P‐tau normalized by NF. Data represents mean + SEM. For each condition, three batches of experiments, one coverslip per batch were analyzed. Two‐way ANOVA. *p < 0.05; ****p < 0.0001.

**FIGURE 3:**
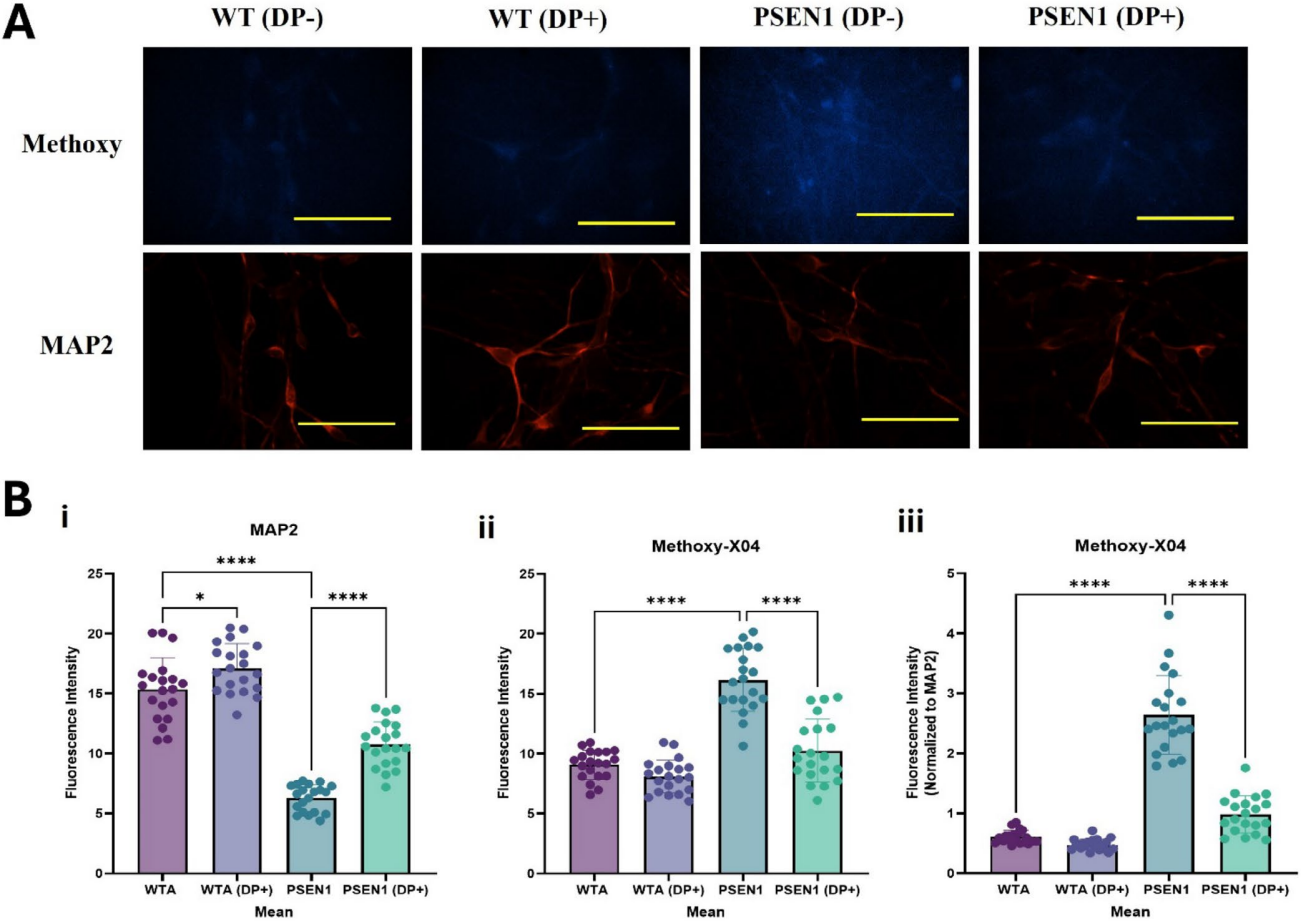
Evaluation of DP effect on Amyloid deposition by Methoxy‐X04 staining after 3 weeks of DP treatment. (A) Confocal images of WT and *PSEN1* CNs stained with MAP2 (red) and Methoxy‐X04 (blue). Scale bar: 100 μm. (B) Quantification of the mean intensity of the images for the signals of: (i) MAP2; (ii) Methoxy‐X04; and (iii) Methoxy‐X04 normalized by MAP2. Data represents mean + SEM. For each condition, three batches of experiments, one coverslip per batch, were analyzed. Two‐way ANOVA. *p < 0.05; ****p < 0.0001.

**FIGURE 4:**
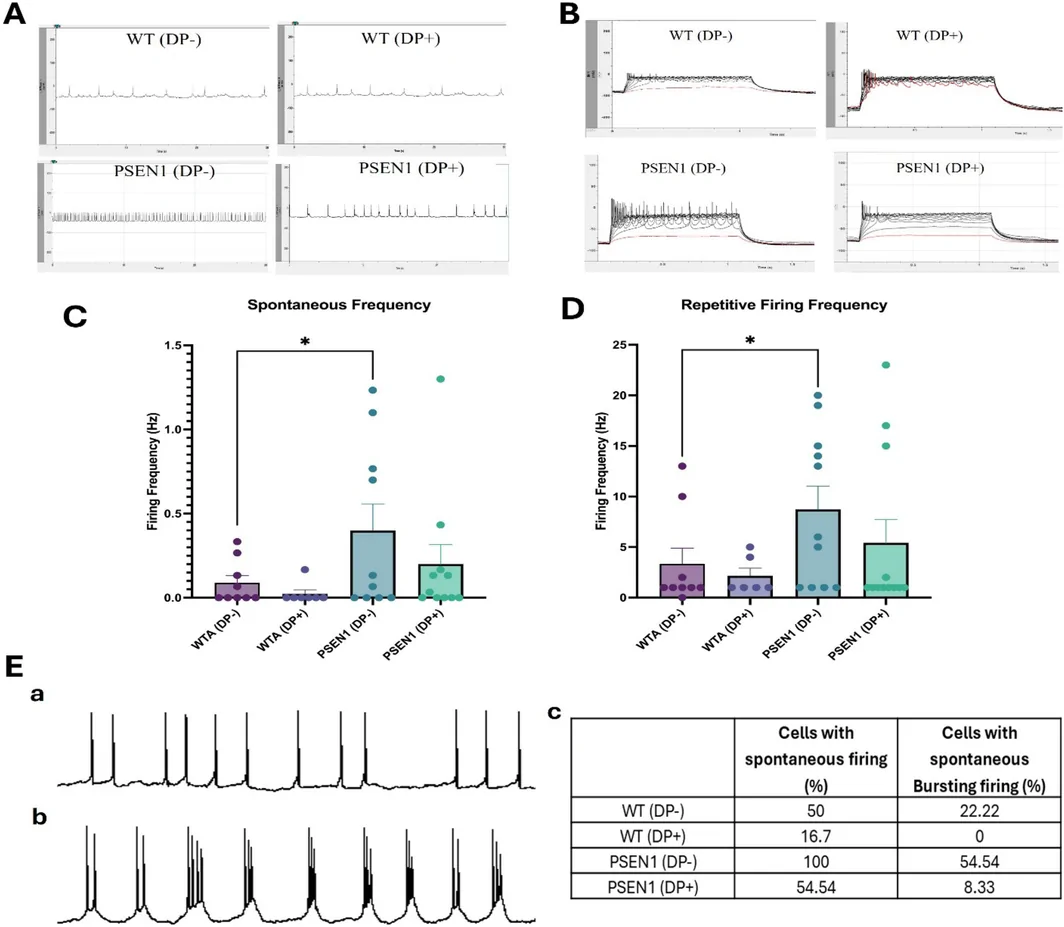
Evaluation of DP effect on cortical neuron electrophysiological property by patch clamp analysis after 1 week treatment on DIV 21 cortical neurons. (A) Sample spontaneous firing activity recorded under gapfree condition from each group. (B) Sample repetitive firing activity recorded under stepwise depolarization from each condition. (C) Mean spontaneous action potential firing frequency. Treatment of DP reduced the frequency in both WT and *PSEN1* CNs but no statistical significance (*p* = 0.1, *p* = 0.08 respectively). (D) Mean repetitive firing frequency quantified. Treatment of DP reduced the frequency in both WT and *PSEN1* CNs but no statistical significance (*p* = 0.26, *p* = 0.16 respectively). Data represent mean + SEM. (E) AP bursting behavior under spontaneous firing frequently observed in *PSEN1* cortical neurons and its inhibition by DP: (a) regular spontaneous firing; (b) bursting AP; (c) quantification of cell percentages demonstrating bursting firing. More than three batches of experiments, 7–12 cells were analyzed for each condition. Student's *t*‐test (unpaired, one‐tailed). *p < 0.05.

**FIGURE 5:**
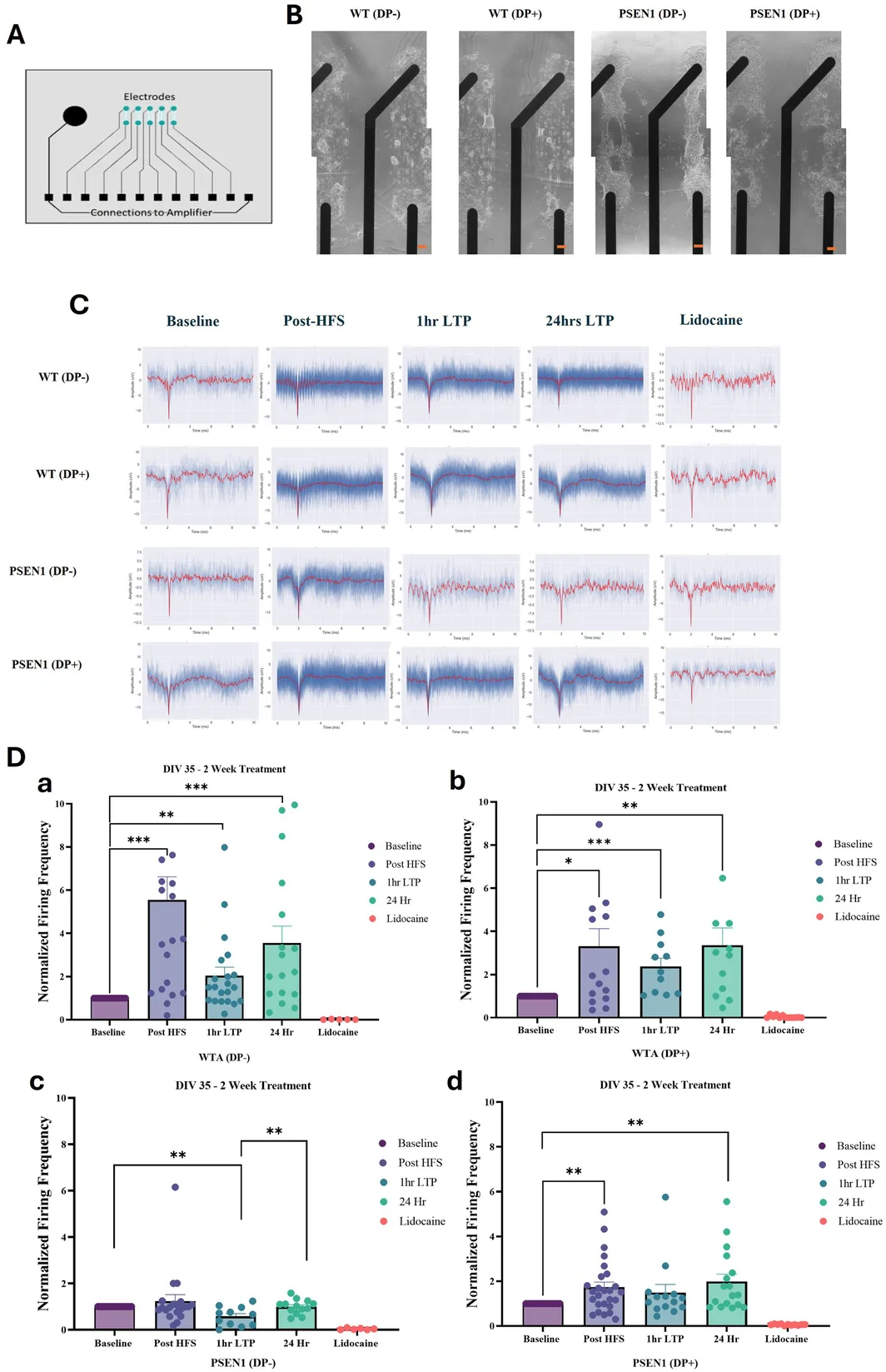
Evaluation of DP effect on neural circuit activity by MEA analysis. (A) Sample image of an MEA chip. (B) Sample images of cortical neural circuits formed on patterned MEAs from each condition. Scale bar: 100 μm. (C) Sample neural spike overlays of cortical neural circuits from each condition. (D) LTP analysis for each experimental group indicating the quantified neural activity at baseline, right after high‐frequency stimulation (post‐HFS), 1 h, and 24 h post‐HFS. (a) WT, (b) WT + DP, (c) *PSEN1*, and (d) *PSEN1* + DP. For each condition, more than three batches of experiments and more than 10 biological replicates were analyzed. Student's *t*‐test (paired, one‐tailed). *p < 0.05; **p < 0.01; ***p < 0.001.

**FIGURE 6:**
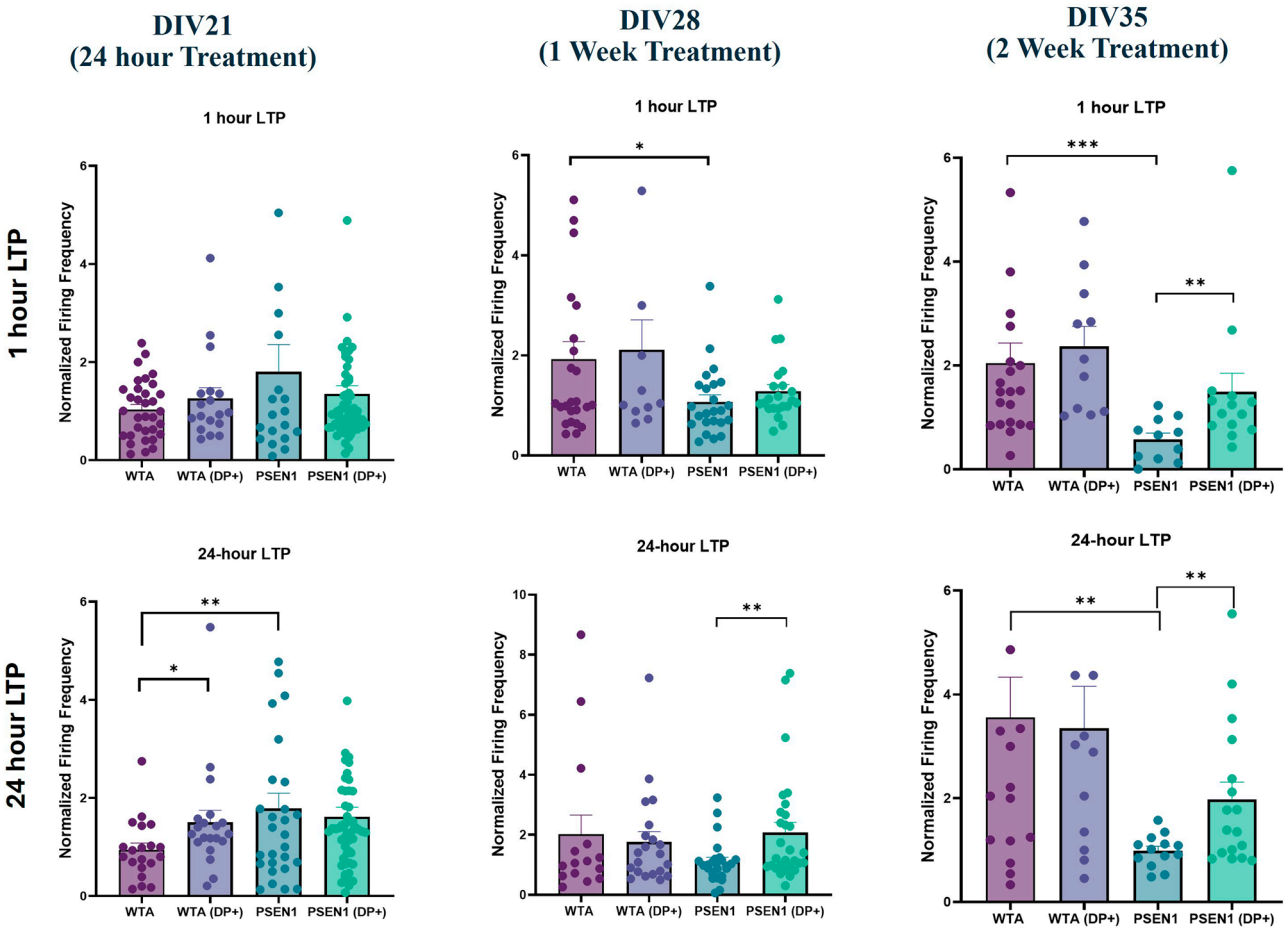
Longitudinal effect of DP on neural circuit activity by MEA analysis. Data demonstrated are mean 1‐h LTP (top panel) and 24‐h LTP (bottom panel), after 24 h (DIV21, left column), 1 week (DIV 28, middle column), and 2 weeks (DIV 35, right column) of DP treatment. Data represents mean + SEM. For each condition, more than three batches of experiments and more than 10 biological replicates were analyzed. Student's *t*‐test (unpaired, one‐tailed). *p < 0.05; **p < 0.01; ***p < 0.001.

Current work can be improved and advanced in future studies. First, the mechanism of the DP's effect on mitochondria function and cell metabolism can be further investigated by measuring MMP with a ratiometric fluorescent analysis (JC‐1 assay) rather than TMRE (a single channel fluorescent assay), mitochondrial function (Seahorse Mito Stress Test), and TCA‐cycle carbon flux (e.g., ^13^C metabolic flux tracing). Astrocytes and/or microglia can be integrated in current neuronal cultures to better recapitulate the cellular property and complexity of the human brain. Additionally, formal blinding of experimental groups can be integrated in future study in experimental design, data collection and analysis.

This research has shown that reintroducing AKG and GABA into AD neurons normalized mitochondria membrane potential, preserved neuronal structure as characterized by the analysis of NF and MAP2, reduced AD pathological markers amyloid and P‐tau, suppressed neuronal hyperexcitability, and re‐establishes neurocircuit synaptic plasticity as manifested by LTP analysis. The DP, composed of AKG, GABA, and arginine in proprietary proportions refined through clinical observation of symptom improvement, leverages the combination of these mechanisms: AKG bypasses the pyruvate blockade, re‐energizing the TCA cycle; GABA restores inhibitory tone and limits excitotoxicity; and arginine, a bipolar base, neutralizes AKG's acidity for oral tolerance while generating NO to enhance microvascular perfusion. The DP represents an anaplerotic therapy by directly addressing the energetic root cause of ND, rather than a downstream disease modifier. Given that bioenergetic insufficiency underlies diverse neurodegenerative pathologies, the DP may provide a unifying metabolic treatment platform. Its components are physiologic and well‐tolerated, facilitating translational application and combination with other mitochondrial or NAD^+^‐enhancing therapies. The convergence of metabolic restoration and electrophysiologic recovery demonstrated in this research supports the principle that energy deficiency is a key driver of neurodegeneration, and that the DP offers a viable and mechanistically grounded path toward its reversal.

## 4 Materials and Methods

### 4.1 HiPSC‐Cortical Neurons Seeding and Maintenance

Cortical neurons differentiated from human iPSCs carrying either a *PSEN1* ^*A246E*^ mutation (purchased from Cedars Sinai) and a WT genotype (ND41865, purchased from Corriell Institute) were differentiated and cultured according to our established protocol [22]. Cryopreserved neurons were recovered in CN maintenance medium and plated onto glass coverslips (200 cells/mm^2^) or MEA (500 cells/cm^2^) pre‐coated with poly‐d‐lysine (0.1 mg/mL), fibronectin (10 μg/mL), and laminin (10 μg/mL). A full‐medium exchange was performed 24 h following cell seeding, after which cells were only maintained by a half‐medium exchange every other day. Starting on Day 7 post‐plating, neuronal cultures were maintained in iPSC‐Cortical Neuron BrainPhys‐based Medium for the duration of the culture at 37°C with 5% CO_2_. Cell maturation stages were tracked by days in vitro (DIV). Details of all the media can be found in [22].

### 4.2 DP Formulation and Treatment

DP powder is a commercially available product from DP (TDP‐AAKG1). Each commercial serving consists of alpha‐ketoglutarate and gamma‐aminobutyric acid within the patented ranges of 3–9 g AKG and 50–140 mg GABA per serving. The precise fixed AKG composition is proprietary. All experiments used the product from the same manufacturing lot. To prepare the DP applicable to the cell culture, the 1000X stock (0.151 mg/mL) was first prepared from the powder in BrainPhys medium, aliquoted, and stored at −20°C. The stock was further diluted into BrainPhys feeding medium (1 μL stock per 1 mL BrainPhys) to yield final 1X working concentration. DP treatment began at DIV14 and was replenished at each regular feeding thereafter by half‐medium change with medium supplemented with 1X DP. The control cultures were given BrainPhys medium at the matched volume. Readouts were collected at 24 h (DIV15), 7 days (DIV21), and 14 days (DIV28) post‐treatment onset.

### 4.3 MEA Recordings

Spontaneous network activity was recorded using an 8‐electrode MEA system (Axion BioSystems). The sampling rate was 12.5 kHz, and spikes were identified using an adaptive threshold of four standard deviations above background noise. Electrode data not experiencing an abolishment of activity following lidocaine addition were excluded from analysis. Data collected from each electrode were normalized to its respective baseline to assess fold changes in activity following HFS and subsequent treatment.

### 4.4 Whole‐Cell Patch‐Clamp Recordings

Electrophysiological properties of single neurons were evaluated using whole‐cell patch‐clamp recording (Axopatch 200B amplifier, Molecular Devices). Recording electrodes (3–5 MΩ) were filled with an intracellular solution containing (in mM): 120 K‐gluconate, 10 KCl, 10 HEPES, 1 EGTA, 4 Mg‐ATP, and 0.3 Na‐GTP (pH 7.3). The extracellular bath solution contained (in mM): 140 NaCl, 3 KCl, 2 CaCl_2_, 1 MgCl_2_, 10 HEPES, and 10 glucose (pH 7.4). In voltage‐clamp mode, neurons were held at −70 mV, and 10 mV step depolarizations elicited Na^+^ and K^+^ currents. Action potential amplitude and repetitive firing frequency were recorded in current‐clamp mode. Data were digitized at 10 kHz and filtered at 2 kHz. Series resistance was compensated to < 20%. Analysis was conducted in Clampfit 10.7 and GraphPad Prism 10. Cells with unstable access resistance were excluded from analysis.

### 4.5 Immunocytochemistry

Cells were fixed with 4% paraformaldehyde for 15 min at room temperature, washed in PBS, permeabilized with 0.1% Triton X‐100 for 10 min, and blocked for 1 h in 5% normal goat serum. Cells were then incubated overnight at 4°C with the following primary antibodies at desired mixture: Chicken‐anti‐MAP2 (Invitrogen, Cat PA1‐10005, 1:1000) and Mouse‐anti‐NF (BioLegend, Cat 837904, 1:500) to assess neuronal morphology, Rabbit‐anti‐Phospho‐Tau (P‐TAUThr181, Cell Signaling, Cat12885, 1:500) to evaluate tau pathology. Methoxy‐04 (M04) (R&D Systems, Cat 4920) (1 μM), when included, was added directly to the primary antibody solution and co‐incubated overnight. Methoxy‐04 is a fluorescent β‐sheet‐binding dye used to detect amyloid‐like aggregates or early fibrillar tau conformers in neuronal cultures. After overnight incubation, cells were washed three times with PBS and incubated with Alexa Fluor‐conjugated secondary antibodies (1:1000) for 1 h at room temperature in the dark. DAPI was applied at 1 μg/mL for 5 min following secondary antibody incubation. DAPI served as a nuclear counterstain for cell counting, morphology assessment, and normalization of fluorescence intensities. Coverslips were mounted using ProLong Gold Antifade reagent.

### 4.6 Imaging and Quantification

Images were acquired on a Zeiss LSM 900 confocal microscope using a ×63 oil‐immersion objective under identical laser, gain, and exposure settings across all groups. Seven images were taken from each coverslip at random but different areas of each coverslip. Quantification was performed in ImageJ (FIJI). P‐TAU fluorescence intensity was normalized to NF to account for neuronal density variation. Methoxy‐04 signal was quantified as total corrected cell‐associated fluorescence (TCCF). DAPI counts were used for normalization when appropriate. Imaging and quantification were performed by the study authors without formal blinding to experimental group; sampling bias was minimized by acquiring images from multiple randomly selected fields per coverslip under standardized acquisition settings as described above.

### 4.7 Statistical Analysis

All analysis was based on experiments of at least three independent experiments. Quantitative data are expressed as mean ± standard error (SEM). Depending on the data content and the aim of analysis, statistical significance between groups was determined using either Student's *t*‐tests for pairwise comparisons or two‐way ANOVA with Tukey's post hoc test for multiple groups. *p* < 0.05 was considered statistically significant. Data visualization and statistical analyses were performed using GraphPad Prism 10.

## Author Contributions

**James J. Hickman**, **Xiufang Guo**, and **Vince Tedone:** conceptualization. **Leticia Keler Soares**, **Gaurav Srivastava**, **Camden Dwelle**, and **Brianna Waggoner:** investigation. **Leticia Keler Soares**, **Xiufang Guo**, and **James J. Hickman:** methodology. **Leticia Keler Soares** and **Gaurav Srivastava:** original data curation. **Xiufang Guo** and **James J. Hickman:** review of data curation. **Leticia Keler Soares** and **Xiufang Guo:** formal data analysis. **James J. Hickman** and **Xiufang Guo:** funding acquisition. **Xiufang Guo** and **James J. Hickman:** experimental guidance. **Xiufang Guo** and **James J. Hickman:** project administration. **Xiufang Guo** and **James J. Hickman:** supervision. **Xiufang Guo** and **James J. Hickman:** validation. **Leticia Keler Soares** and **Xiufang Guo:** visualization. **Leticia Keler Soares:** writing – original draft. **Xiufang Guo**, **Leticia Keler Soares**, **Vince Tedone**, and **James J. Hickman:** writing – review and editing.

## Funding

This work was funded by the Winning the Fight Against Neurodegenerative Diseases (WFND) AWD4941; National Institutes of Health, National Institute on Aging grant numbers 1R03AG070514‐01, R44AG071386‐01A1, 1R01AG077651 and 1R43 AG060886‐01.

## Ethics Statement

The human cells to be utilized on this project are all de‐identified and collected/stocked under ethical guidelines by established organizations, and are available to the general research community. All these would fall under NIH Exemption 4 as stated in that the research is not research involving human subjects and 45 CFR part 46 does not apply.

## Consent

All authors consent the publication of this paper.

## Conflicts of Interest

James J. Hickman is the Chief Scientist and member of the Board of Directors of Hesperos Inc. Vince Tedone is a co‐founder and the pro‐bono medical director of the non‐profit organization WFND. The other authors declare no conflicts of interest.

## Supporting information


**Figure S1:** Evaluation of DP effect on cortical neurons (DIV21) axonal morphology by immunocytochemistry analysis after 1 week of DP treatment. Quantification of the images for the signals of: (A) NF; (B) P‐tau; (C) P‐tau normalized by NF. Data represents mean + SEM. More than three batches of experiments were analyzed for each condition. Two‐way ANOVA. *p < 0.05; **p < 0.01; ***p < 0.001; ****p < 0.0001.
**Figure S2:** Sample traces of whole‐cell patch‐clamp recordings of iPSC‐derived cortical neurons for the major parameters analyzed. (A) Sample spontaneous firing activity recorded under gap‐free current‐clamp conditions from each condition. (B) Sample Na^+^/K^+^ membrane currents recorded in voltage‐clamp mode. (C) Sample repetitive firing activity recorded under stepwise current injection. (D) Sample action potential waveform.
**Figure S3:** Quantification of electrophysiological parameters that were not significantly altered by genotype or DP treatment in DIV21 cortical neurons after 1‐week treatment. (A) Action potential. (B) Resting membrane potential (RMP). (C) Membrane resistance. (D) Membrane capacitance. (E) Peak inward Na^+^ current. (F) Peak outward K^+^ current. Data represent mean ± SEM. More than three batches of experiments were analyzed for each condition. Two‐way ANOVA and Tukey's post hoc analysis, *p < 0.05, **p < 0.01, ***p < 0.001, ***p < 0.0001.
**Figure S4:** Evaluation of DP effect on DIV 21 neural circuit activity by LTP analysis on MEA. (A) Sample traces of spike overlays for each experimental group indicating the quantified neural activity at baseline, right after high frequency stimulation (HFS), 1 h, and 24 h post‐HFS. (B) Quantification of spike overlays at different time points. (a) WT, (b) WT + DP, (c) PSEN1, (d) PSEN1 + DP. DP treatment potentiated network function in which the increase in post‐HFS, 1‐h, and 24‐h LTP became statistically significant. More than three batches of experiments were analyzed for each condition. Two‐way ANOVA. *p < 0.05; **p < 0.01; ***p < 0.001.
**Figure S5:** Evaluation of DP effect on DIV 28 neural circuit activity by LTP analysis on MEA. (A) Sample traces of spike overlays for each experimental group indicating the quantified neural activity at baseline, right after high frequency stimulation (HFS), 1 h post‐HFS, and 24 h post‐HFS. (B) Quantification of spike overlays for each experimental group at different time points. (a) WT, (b) WT + DP, (c) PSEN1, (d) PSEN1 + DP. One week of DP treatment significantly induced increase of both 1 h and 24 LTP in both WT and *PSEN1* systems. More than three batches of experiments were analyzed for each condition. Two‐way ANOVA. *p < 0.05; **p < 0.01; ***p < 0.001; ****p < 0.0001.

## Data Availability

Data is provided within the manuscript or Supporting Information files. The datasets are available from the corresponding author on reasonable request.
