# Supplementary material for: Deanna Protocol Ameliorates the Deficits in Human iPSC‐Cortical Neurons Carrying Familial Alzheimer's Mutation
Source: CNS Neurosci Ther. 2026 Oct 23;32(10):e71205. doi: 10.1002/cns.71205 (PMC13691636; doi:10.1002/cns.71205)
Supplement: Supplementary file 1 [file CNS-32-e71205-s001.docx]

**Supplementary Figure S1. Evaluation of DP effect on cortical neurons (DIV21) axonal morphology by immunocytochemistry analysis after 1 week of DP treatment.** Quantification of the images for the signals of: **A)** NF; **B)** P-tau; **C)** P-tau normalized by NF. Data represents Mean+ SEM. More than 3 batches of experiments were analyzed for each condition. Two-way ANOVA. ****, P<0.0001.

**Supplementary Figure S2.** **Sample traces of** **whole-cell patch-clamp recordings of iPSC-derived cortical neurons for the major parameters analyzed.** **A)** Sample spontaneous firing activity recorded under gap-free current-clamp conditions from each condition. **B)** Sample Na⁺/K⁺ membrane currents recorded in voltage-clamp mode. **C)** Sample repetitive firing activity recorded under stepwise current injection. **D)** Sample action potential waveform.

**Supplementary Figure S3.** **Quantification of electrophysiological parameters that were not significantly altered by genotype or DP treatment in DIV21 cortical neurons after 1-week treatment. A)** Action potential. **B)** Resting membrane potential (RMP). **C)** Membrane resistance. **D)** Membrane capacitance. **E)** Peak inward Na⁺ current. **F)** Peak outward K⁺ current. Data represent Mean ± SEM. More than 3 batches of experiments were analyzed for each condition. Two-way ANOVA Two-Way ANOVA and Tukey’s post-hoc analysis.

**Supplementary Figure S4. Evaluation of DP effect on DIV 21 neural circuit activity by LTP analysis on MEA. A)** Sample traces of spike overlays for each experimental group indicating the quantified neural activity at baseline, right after high frequency stimulation (HFS), 1 hour and 24 hours post-HFS. **B)** Quantification of spike overlays at different time points. a) WT, b) WT+DP, c) PSEN1, d) PSEN1 + DP. DP treatment potentiated network function in which the increase in post-HFS, 1-hr and 24-hr LTP became statistically significant. More than 3 batches of experiments were analyzed for each condition. Two-way ANOVA. *, P<0.05; **, P<0.01; ***, P<0.001.

**Supplementary Figure S5. Evaluation of DP effect on DIV 28 neural circuit activity by LTP analysis on MEA. A)** Sample traces of spike overlays for each experimental group indicating the quantified neural activity at baseline, right after high frequency stimulation (HFS), 1 hour post-HFS, and 24 hours post-HFS. **B)** Quantification of spike overlays for each experimental group at different time points. a) WT, b) WT+DP, c) PSEN1, d) PSEN1 + DP. One week of DP treatment significantly induced increase of both 1hr and 24 LTP in both WT and *PSEN1* systems. More than 3 batches of experiments were analyzed for each condition. Two-way ANOVA. *, P<0.05; **, P<0.01; ***, P<0.001; ****, P<0.0001.

**Supplementary Figure S1.**

**Supplementary Figure S2.**

**Supplementary Figure S3.**

**Supplementary Figure S4.**

**Supplementary Figure S5.**
